# Acknowledgment to the Reviewers of *Vaccines* in 2022

**DOI:** 10.3390/vaccines11020227

**Published:** 2023-01-19

**Authors:** Vaccines Editorial Office

**Affiliations:** MDPI AG, St. Alban-Anlage 66, 4052 Basel, Switzerland

High-quality academic publishing is built on rigorous peer review. *Vaccines* was able to uphold its high standards for published papers due to the outstanding efforts of our reviewers. Thanks to the efforts of our reviewers in 2022, the median time to first decision was 18 days and the median time to publication was 39 days. Regardless of whether the articles they examined were ultimately published, the editors would like to express their appreciation and thank the following reviewers for the time and dedication that they have shown *Vaccines*:
Aamodt, GeirLim, Siew PhengAbbasi Oshaghi, EbrahimLim, Theam SoonAbboubakar, HamadjamLim, Weng MarcAbdallah, AtiyehLima, Julie C.Abdel Moneim, Ahmed E.Limbu, YamAbdelgawad, MohamedLimongi, JeanAbdel-LAtif, Hany M. R.Lin, CailuAbdoli, AmirLin, CarolAbedini, AtefehLin, Chang-ChiAbilash, Valsala GopalakrishnanLin, Cheng-WenAbouelhadid, SherifLin, Chung-YingAbraham, RachyLin, Jin-DingAbreu, Celina MonteiroLin, XiaorongAbudabos, Alaeldein M.Linial, MichalAcanfora, DomenicoLinz, BodoAdadi, PariseLio, DomenicoAdamo, RobertoLirette, SethAdamovicz, Jeffrey J.Lis Arias, Manuel J.Adan, AnaLiu, BoyaAddie, DianeLiu, DiAdepoju, OmololaLiu, HaipengAdjobimey, TomabuLiu, HaolinAdnan, MohdLiu, HaopingAdriawan, Ignatius RyanLiu, HejunAdunyah, Samuel E.Liu, JiaAfrin, FarhatLiu, KefangAgarwal, AniruddhaLiu, LihongAggarwal, SomyaLiu, QianAgha, SohailLiu, QiangAgmon-Levin, NancyLiu, QingyunAguilar, Julio CésarLiu, Shih-JenAguinaga-Ontoso, InésLiu, SufangAherfi, SarahLiu, XinranAhmad, GulLivaniou, EvangeliaAhmad, JavedLizano, MarcelaAhmad, KhurshidLlopis-González, AgustínAhmad, SajjadLo, ChristopherAhmadian, GholamrezaLo, Shih-YenAhmed, Shaimaa A. A.Loglio, AlessandroAhmed, TofayelLogoyda, Liliya S.Ahn, JeonghyunLohmann, Katharina L.Aimanianda, VishukumarLondero, Ambrogio P.Aithal, AbhijitLong, BradleyAkbar, Sheikh Mohammad FazleLong, CarrieAkbulut, SamiLonget, StephanieAkova, MuratLongoni, Silvia StefaniaAktas, MunirLópez, CeferinoAktaş, OsmanLopez-Labrador, Francesc-XavierAl Awaidy, SalahLorenzen, NielsAl Basir, FahadLorusso, AlessioAladaǧ, MuratLorusso, FeliceAlagarasu, KalichamyLoubet, PaulAlaimo, AlessandroLu, JunfengAl-Alem, UmaimaLu, MaolinAlam, IntikhabLu, ShaoyongAlam, KhurshidLucenteforte, ErsiliaAlam, SaminaLuk, Hsiang-NingAlameh, MohamadLukas, MilanAlbahri, Ahmed ShihabLukashevich, IgorAlbendín-García, LuisLukhwareni, AzwidowiAlberto, MacchiLukov, GeorgiAlbrecht, Don E.Luna Maldonado, AurelioAlbrecht, RandyLundstrom, KennethAlcolea, Pedro J.Lunel-Fabiani, FrançoiseAlderson, MarkLuo, RayAldous, ColleenLurie, NicoleAl-ebini, YousefLusher, Joanne MarieAleksova, AnetaLüth, StefanAlexander-Miller, Martha A.Luttermann, ChristineAlgeo, Timothy P.Lv, HuibinAlghamdi, SaadLysyk, TimothyAlhamo, MoyasarŁyszkiewicz, MarcinAli Abumalloh, RababLytton, SimonAli, IshtiaqLyu, MengzeAli, ShakirMa, HongmingAli, Sheikh TaslimMacaluso, GiusiAlishetty, SumanMaccagno, MassimoAlkan, MichaelMacdonald, Noni E.Al-Karmalawy, Ahmed A.MacEwan, Sarah R.Allain, Jean-PierreMachado, Luis FernandoAl-Lami, FarisMacLeod, David A.Allegaert, KarelMaeda, AkihikoAllen, DavidMaeda, KazuhikoAllen, David M.Maeda-Chubachi, TomokoAlloni, RossanaMaffini, FaustoAloysius, IvanMagagnoli, FedericaAltomonte, JenniferMagariños Ferro, BeatrizAltunay, İlknur KıvançMagiorkinis, Emmanouil N.Alugupalli, KishoreMaglietti, Felipe HoracioÁlvarez-Mercado, Ana I.Magnaldo, ThierryAlvarez-Sanchez, María ElizbethMagnavita, NicolaAlving, KjellMagori, KrisztianAly, Ahmed S. I.Magrone, TheaAmarante, Alessandro F. T.Mahalingam, Shanmuga S.Amarilla, Alberto A.Mahanty, SiddharthaAmaro-Estrada, ItzelMahapatra, DurgaAmman, BrianMaharani, AsriAmodio, DonatoMahdi, LaylaAmodio, EmanueleMahfouz, Mohamed SalihAmu, SylvieMahmutefendić Lučin, HanaAn, DongMahony, Timothy JohnAn, Seong Soo A.Maiese, AnielloAnahtar, Melis N.Maietti, ElisaAnaniev, JulianMainou, BernardoAnantpadma, ManuMajewska, AnnaAnastasopoulos, EleftheriosMajima, Hideyuki J.Anatskaya, OlgaMajlessi, LalehAnbarasu, AnandMak, Gannon C. K.Anderson, Christopher S.Mak, Lung-YiAndo, TomoakiMalcangi, GiuseppinaAndonov, AntonMaldonado, EdioAndrade, Heitor, Jr.Malhotra, PawanAndrew, MacintyreMalinauskas, RomualdasAngastiniotis, MichaelMaŁkowski, Piotr MirosławAngelucci, Clotilde B.Mallagaray, AlvaroAnifandis, GeorgeMallhi, Tauqeer HussainAnstead, Gregory M.Maloberti, AlessandroAnticoli, SimonaMaltezou, Helena C.Antipova, Veronica AlexandraMan, Siu ShingAntonelli, AlbertoManam, SrikanthAntonelli, ElisabettaMancon, AlessandroAntoniu, Sabina AntonelaMandić-Rajčević, StefanAnusevičius, ŽilvinasMandilara, GeorgiaArai, SatoruManelli, FilippoAraújo, Eugênio Gonçalves deManetti, Alice ChiaraArcangeli, GiulioManfellotto, DarioArcari, LucaManfredi, Maria TeresaArciniega, JuanMannan, Kazi AbdulArciszewski, Marcin BartłomiejManne, KartikAriani, AlaricoManni, GianlucaAricò, MaurizioManso, Martina DelAriga, KatsuhikoMantri, Chinmay KumarArmengot-Carceller, MiguelManzoor, RashidArmesto Alonso, SusanaMara, ArlindArminen, IlkkaMarc, DanielArmstrong, Mark RobinMarchesi, FedericoArnalich, FranciscoMarcotrigiano, JosephArnedo-Pena, AlbertoMarczyńska, MagdalenaArnhold, StefanMaree, Francois F.Arokiyaraj, SelvarajMargina, DenisaArora, GunjanMaría Compañ, LauraArranz-Solís, DavidMarichannegowda, Manukumar HonnayakanahalliArrieta, AntonioMarimon, Jose MariaArunachalam, MurugeshMarin Lopez, AlejandroAsad, MohammedMarinelli, EnricoAsakawa, MitsuhikoMaringhini, SilvioAsare, MattMarinos, GeorgiosAsaro, FiorettaMarion, Jason W.Ashino, YugoMarkopoulos, AnastasiosAshiru-Oredope, DianeMarongiu, FrancescoAshraf, Ghulam MdMarotta, ClaudiaAskling, Helena HerviusMarracci, SilviaAsproni, PietroMarriott, AnthonyAsten, HenriMarruchella, GiuseppeAtanasova, KalinaMarsilio, FulvioAthanassiou, PanagiotisMarson, Fernando Augusto LimaAthina, PyrpasopoulouMartin, PatriciaAttia, SamehMartin, Rebecca K.Attia, YoussefMartín-Cófreces, Noa B.Aubert, RachaelMartinez, MagalyAugustyniak, DariaMartinez, OsvaldoAung, Meiji SoeMartínez-Beneyto, YolandaAvirutnan, PanisadeeMartinez-Jarreta, BegoñaAw, Tar ChoonMartínez-López, José ÁngelAydede, Sema K.Martínez-Navarrete, GemaAzab, WalidMartínez-Varea, AliciaAzad, AbduMartin-Galiano, Antonio J.Azevedo, AnaMartín-García, ElenaAzevedo-Silva, JoãoMartino, Piera AnnaAzzari, ChiaraMarzuillo, CarolinaAzzi, AlbertaMasa’deh, Ra’edBabitsch, BirgitMasachessi, GiselaBabiuk, ShawnMasarone, DanieleBaccolini, ValentinaMasavuli, MakutiroBachmann, Martin F.Mascarenhas, MayaBadiola, IgnacioMascia, GiuseppeBahri, RajiaMasoller, NarcísBahrs, ChristinaMason, PaolaBaicus, CristianMassoni, FrancescoBailey, Adam LeeMastrangelo, PeterBaindara, PiyushMasujin, KentaroBaiocchi, Robert A.Matanock, AlmeaBaker, Steven F.Mateos-Hernández, LourdesBakiu, RigersMatheu, VictorBakre, AbhijeetMathew, SijoBalaraman, VelmuruganMathioudakis, Alexander G.Balieva, Flora N.Matiašovic, JánBallini, AndreaMatsuda, YoshikoBaloch, ZulqarnainMatsui, MasanoriBanach, KlaudiaMatsukuma, SusumuBandara, Aloka B.Matsumoto, KazuhikoBanday, Abid H.Matsumoto, YasuhikoBandyopadhyay, DhrubajyotiMatsuo, MuneakiBankala, KrishnarjunaMatsuzaka, YasunariBánki, ZoltánMatteucci, ClaudiaBanoub, Joseph H.Mattiuzzo, GiadaBansal, KushagraMattner, JochenBao, YongmingMatuszak, MagdalenaBarák, ImrichMatveeva, OlgaBaraniak, AnnaMaugeri, Andrea GiuseppeBarbas, Carmen Sílvia ValenteMauriz, ElbaBarba-Spaeth, GiovannaMaurya, Vineet KumarBarberis, ElettraMaves, Ryan CoryBar-Haim, ErezMavrogonatou, EleniBarletta, Raul G.Mayer, Rupert L.Barman, SoumikMayner, LidiaBarnard, MarieMays, Jody K.Barnett, BradMazor, OhadBar-On, YotamMazurov, Dmitriy V.Barroso, HelenaMazzoni, AlessioBaršić, BrunoMbonye, UriBartholomeeusen, KoenMcCarthy, Katharine J.Bartosińska, JoannaMcElroy, JaneBartůňková, JiřinaMcGrew, MikeBasheeruddin, Syed MohammedMcIntosh, E. David G.Basso, MonicaMcKinstry, Karl KaiBastian, MaxMcVey, DavidBastos, Reginaldo G.Medić, SnežanaBasyuni, MohammadMedina, Gisselle N.Bates, John T.Meena, JairamBatra, HimanshuMegawati, DewiBatra, KavitaMehmood, RashidBatra, LalitMei, HanBaud, StephanieMei, Kuo-ChingBäumer, WolfgangMejía-Aranguré, Juan ManuelBaur, CynthiaMekkawy, Ahmed H.Bauwens, JorgenMella, AlbertoBavananthasivam, JegarubeeMellou, KassianiBavari, SinaMencacci, AntonellaBawage, SwapnilMendez-Lagares, GemaBayart, Jean-LouisMendonça, Elismauro FranciscoBayer, WibkeMeneguzzo, PaoloBeard, FrankMeneses, PatricioBeckham, J. DavidMenzo, StefanoBedolla-Barajas, MartínMercado, Noe B.Beemon, KarenMerino, Jose JoaquinBegum, AnjumanMertig, AngelaBehravesh, Casey BartonMeseda, ClementBeilby, JustinMesquita, JoãoBélec, LaurentMessina, FrancescoBellini, SilviaMessina, GabrieleBellomo, NicolaMestres, ConxitaBeloukas, ApostolosMestrovic, TomislavBelov, GeorgeMetifiot, MathieuBeltowski, JerzyMeulebroeck, WendyBenbow, EmyrMeurice, FrançoisBenchimol, MarleneMeyer, BenjaminBencurova, ElenaMeyer, HermannBéné, Marie C.Meyer, Michelle N.Benevene, PaulaMeyer, SaschaBenfield, David A.Meyers, CraigBenis, ArrielMeysamie, AlipashaBenites-Zapata, Vicente A.Mezei, MihalyBenitez, Gabriela VazquezMichael Samarkos, MichaelBenkő, RiaMichaelis, MartinBeran, JiriMichail, Georgios D.Beran, RoyMicheli, ValeriaBerar-Yanay, NoaMigliore, EnricaBerber, EnginMigliorini, PaolaBerçot, BeatriceMikolajczyk, AgataBercovier, HerveMilani, Gregorio PaoloBerezin, AlexanderMilano, FrancescaBergh, ØivindMilasin, JelenaBergqvist, AndersMilavetz, BarryBerguer, Paula M.Milazzo, LauraBerkman, Samuel A.Mileva, MilkaBerlina, Anna N.Miller, ElizabethBerlit, PeterMing, Long ChiauBernardi, SaraMir, JanBertellini, ElisabettaMirza, ImranBerth, HendrikMirzaei, RasoulBertho, NicolasMisaki, RyoBerti, FrancescoMishto, MicheleBertolotti, LuigiMiśkiewicz, PiotrBertram, MirandaMiteva, Lyuba DinevaBertzbach, Luca DaniloMitra, SaikatBerzero, GiuliaMitrakas, AchilleasBestetti, Reinaldo B.Mitsuhashi, ToshiharuBetakova, TatianaMittal, AnshumaliBettencourt, PauloMittas, NikolaosBeyer, KatharinaMiwa, KunihisaBezos, JavierMiyazawa, KohtaroBhagavathula, Akshaya SrikanthMizukami, ShusakuBhalla, ManmeetMocenni, ChiaraBhargava, AditiModenese, AlbertoBhattacharjee, MrinalMoe, Gregory R.Bhawal, Ujjal KumarMoffat, Jennifer F.Bhosle, Sushma M.Mohamed, WaleedBhowmik, DebipreetaMohamed-Hussein, Aliae A. R.Bianchi, FabrizioMohsen, MonaBianchi, Francesco PaoloMohsin, MuhammadBianchi, SerenaMojumdar, KamalikaBianco, AidaMolinaro, SabrinaBianco, FrancescoMöller, SörenBibi, ShabanaMomi, StefaniaBicout, DominiqueMomin, ZeineenBieganowski, PawelMomtaz, Yadollah AbolfathiBielefeldt-Ohmann, HelleMonget, PhilippeBiering, Scott B.Mongin, DenisBiernasiuk, AnnaMonif, GillesBilbao, DanielMoniuszko, AnnaBilliald, PhilippeMonroy, FernandoBilyy, RostyslavMontazeri, AliBiondi, VitoMontisci, MassimoBirau, Felicia RamonaMontuori, NunziaBirge, Raymond B.Moon, Hyeun JunBirhan, Yihenew SimegniewMor, SunilBisping, GuidoMora, Delio JoséBizzarri, RanieriMorales Suárez-Varela, María M.Björkbacka, HarryMorales, AlejandroBlanco, JoséMorales-Lange, ByronBlasco, RafaelMoreira, Ana Maria Da PalmaBoccalini, SaraMoreno, EstherBocedi, AlessioMoretti, FrancescaBogucki, JacekMoretti, SoniaBøgwald, JarlMori, AntonioBöhler, ThomasMori, MasaakiBöhmer, Merle M.Moroi, MasaoBohmwald, KarenMorozova, OlgaBolboaca, SoranaMorris, Stephen A.Bolcato, MatteoMorrone, PietroBoletis, IoannisMosa, Alexander I.Bonay, MarcelMosbah, RashaBonetti, GraziellaMoshammer, HannsBongiovanni, MarcoMotola, DomenicoBonini, SergioMouchtouri, Varvara A.Bono, María RosaMould-Quevedo, Joaquin F.Bonsignore, AlessandroMoulick, AmitavaBonyah, EbenezerMoura, Liane I. F.Boonnak, KobpornMourão, Paulo ReisBoquet, DidierMpendulo, Magagula VusiBoratto, PaoloMphekgwana, Peter ModupiBorchiellini, AlessandraMrugalska, BeataBorkakoty, BiswajyotiMu, YunxiangBorkow, GadiMu, ZekunBoroń-Kaczmarska, AnnaMucker, Eric M.Boros, Laszlo G.Muhammad, KhalidBorras, EvaMujtaba, HasanBorsuk, SibeleMukherjee, AnupamBoshra, Hani Y.Mukherjee, JeanBostik, PavelMukherjee, RatnadeepBotgros, RaduMuller, IlariaBoucau, JulieMüller, Janis A.Bouin, AlexisMüller, UweBoulton, StephenMuller, Yunhua LiBourry, OlivierMunder, AntjeBoussarsar, MohamedMuniraju, MuraliBoussios, StergiosMuñoz Bellido, Juan LuisBouzid, DoniaMurale, Dhiraj P.Bowen, RichardMurata, ShiroBozic, JovanaMurdaca, GiuseppeBracco, EnricoMurmura, FedericaBradford, DanaMurray, NikiBragazzi, Nicola LuigiMuthupandian, SaravananBraha, AdinaMutinelli, FrancoBrambilla, AndreaMutoloki, StephenBranca, Jacopo Junio ValerioMuttil, PavanBrandt, CurtisMutz, PascalBrantsæter, Arne BrochMwangi, William N.Brasel, TrevorMylonas, EfstratiosBravo, Susana B.N’Guyen, YohanBreban, RomulusNabavi, Seyed MassoodBreiman, AdrienNaeem, AsifBrenner, Michael J.Nagasaka, KazunoriBressollette, CelineNagata, ToshiBrin, MitchellNagelkerke, NicoBrinkmann, FolkeNaik, Abani KantaBrites-Neto, JoséNaik, Parvaiz Ahmad Britt, WilliamNaing, Zin W.Brocchi, MarceloNair, AnroopBrooks, Benjamin D.Najafi Fard, SaeidBrotons, MariaNakakita, Shin-ichiBroukhanski, George C.Nakano, Eduardo YoshioBrown, MarthaNakayama, TetsuoBrown, Richard J. P.Nalca, AysegulBrufau, JoaquimNaldiga, SpandanaBrugnera, EnricoNallasamy, PalanisamyBruns, Anke H. W.Nam, TaewooBruzzese, DarioNamazova-Baranova, Leyla S.Buchhorn, ReinerNante, NicolaBuchta, ChristophNanthapisal, SiraBudzynska, SylwiaNapoli, ChristianBuendía, AntonioNapolitano, FrancescoBugarin, AlejandroNarzisi, AntonioBujnowska-Fedak, Maria MagdalenaNasarre, PatrickBulati, MatteoNashar, TouficBulatov, EmilNashwan, Abdulqadir J.Buonomo, Antonio RiccardoNasti, Tahseen H.Burgess, GrahamNava, VictorBurián, KatalinNavarro-Alvarez, NaluBurnett, EleanorNavarro-Cuenca, AnnaBurns, CarolNawabi, AttaBurton, ZacharyNayrac, ManonButch, Christopher J.Naz, AnamButkauskas, DaliusNazzaro, GianlucaButt, Muhammad HammadNeef, AlexanderButt, Zahid AhmadNeeli, Praveen KumarButu, MarianNees, MatthiasByun, JonghoeNegi, ArvindCaballero, PabloNeglia, GianlucaCabaret, JacquesNejad, Amir Sasan MozaffariCabeda, José ManuelNellimarla, SrinivasCacciola, Emma C.Nelson, Chase W.Cacciola, FrancescoNelson, PatrickCacciola, RossellaNemerow, GlenCada, RomanNémeth, PéterCadeddu, ChiaraNeter, EfratCaenazzo, LucianaNetesov, SergeyCaffò, AlessandroNg, Qin XiangCaggiano, GiuseppinaNganou-Makamdop, KrystelleCain, Derek W.Nguyen, Phuong-HienCăinap, CălinNiazi, Sarfaraz K.Çaki, CanerNicolai, EleonoraCalagna, GloriaNicoletti, LoredanaCalahorra, LeticiaNicoli, FrancescoCaldarola, PasqualeNiederwieser, DietgerCalina, DanielaNiedziela, TomaszCalisti, RobertoNielsen, Vance G.Callendret, BenoitNiemczyk, MariuszCalogiuri, GianfrancoNienhaus, AlbertCamero, MicheleNieuwenhuizen, NatalieCameron, EmilieNigam, ManishaCampa, Carlo CosimoNikbakht Nasrabadi, AlirezaCampise, MariarosariaNikitopoulou, IoannaCampobasso, CarloNiklasson, BoCamus-Bouclainville, ChristelleNikolich, MikeljonCandelli, MarcelloNilubol, DachritCandido, SaverioNinchoji, TakeshiCanè, StefaniaNishizono, AkiraCao, DianjunNissapatorn, VeeranootCao-Lormeau, Van-MaiNistal-Villán, EstanislaoCapobianchi, MariaNitika Capoluongo, NicolinaNitta, TakayukiCappellano, GiuseppeNitulescu, MihaiCaputo, AntonellaNitzan, DoritCarbajo-Lozoya, JavierNjie-Mbye, Ya FatouCarbone, LuigiNoda, HiroyukiCardillo, FabíolaNogueira, AntónioCarmona-Ribeiro, Ana M.Noh, Susan M.Carneiro, LuizNolting, Jacqueline M.Caroleo, Maria CristinaNoor, RashedCarreras, JoaquimNovikov, Alexander S.Carsote, MaraNowak, GlenCartelle Gestal, MonicaNowakowska, ElżbietaCarvalho, EdmundNowalk, Mary PatriciaCasado, José LuisNowsheen, SomairaCasella, GiovanniNtziora, FotinieCasey, TristanNunez, AlejandroCasigliani, VirginiaNuraini, NuningCaspari, ThomasNygård, StåleCassimos, DimitriosNyström, Sofia N.Castañeda, Javier Eduardo GarciaO’Connor, MeganCastaño-Osorio, JhonO’Reilly, MaryCastells, MatíasOancea, CristinaCastiglia, PaoloOberle, Doris F.Caterina Turco, MariaObermann, Ellen ChristinaCavalcanti, Luciano Pamplona De GóesObermeier, PatrickCavann, LuigiObiri-Yeboah, DorcasCaviglioli, GabrieleOdone, AnnaCenter, Rob J.Oğuzoğlu, Tuba ÇiğdemCento, ValeriaOh, HelenCerutti, AndreaOh, Ki-KwangCervantes, JorgeOjanen, Markus J. T.Cerwenka, HerwigOkamoto, MarikoChaari, AliOkamoto, ShigefumiChaicumpa, WanpenOkamoto, YoshinoriChakraborti, SoumyanandaOkamoto, YuriChakraborty, SagnikOkay, SezerChambers, ThomasOkazaki, KatsunoriChan, Yi HaoOkinaka, KeijiChandra, SharatOkumura, KoChaney, CassandraOlaimat, Amin N.Chang, Chee-JanOldfield, NeilChang, Chung-MingOlga Lidia, Vera-LastraChang, Jer-MingOlick, Robert S.Chang, Junn-LiangOliver, StefanChang, Kwang PooOlivera, MarthaChang, SukkumOlotu, FisayoChang, XinyueOlsen, DanielChao, Day-YuOng, EdisonChappell, KeithOno, TakashiCharles Richard, John LalithOo, AdrianCharoentanthanakul, WasinOomens, Antonius (Tom)Chase, P. BryantOpenshaw, PeterChatterjee, SampurnaOrban, ErikaChaudhari, JayeshbhaiOrnello, RaffaeleChaudhry, BeenishOrosz, FerencChaudhuri, MinuOrtega Rodríguez, CarmeloChaudhury, SusmitnarayanOrtega, Hugo H.Chavan, Sandip N.Ortega, Jose AntonioChávez-Galán, LeslieOrtega-Cerrilla, María EstherChawla Sarkar, MamtaOrtiz-Masiá, María DoloresChawla, MohitOrzalesi, NicolaChebloune, YahiaOshansky, ChristineChen, Chih-ShouOsuna, EduardoChen, ChuanOtani, NaruhitoChen, Der-YuanOtani, NorioChen, GuobingOtsuka, NaoChen, HuiOuattara, Eric N.Chen, Kao-ChinOuyang, WeimingChen, MingfuOwais, MohammedChen, MingyiOwusu, DanielChen, Tai-HoOyekale, AbayomiChen, WangxueOyelade, TopeChen, WeiOzaki, ShozoChen, XiangyongOzaslan, MehmetChen, XinyuanPace, LorenzoChen, Yih YuanPaciello, IdaCheng, KuiPadrós, FrancescCherstvy, AndreyPagani, MauroChetta, MassimilianoPagano, StefanoChiang, Shu-ChiungPagnotti, Gabriel M.Chiappori, FedericaPahar, BapiChido-Amajuoyi, Onyema GregPaim, KennioChies, JoséPainter, GavinChinta, Krishna C.Palazzolo, LucaChiou, Shiow-HerPalladino, RaffaeleChivu-Economescu, MihaelaPallua, Johannes DominikusCho, Young-HoPalma, CarlaChodick, GabrielPalma, MarcoChoi, Chang WonPampalakis, GeorgiosChoi, Cheuk-Wai HoracePamučar, DraganChoi, MiraPan, XiaolingChoi, Mi-RanPanasiewicz, GrzegorzChoi, Won HyungPanda, PritamChoi, Won SukPandak, NenadChoi, Youkyung H.Pandey, RajanChoi, Youn-JinPandey, RajeevChoudhary, Om PrakashPandey, RajeshChoumet, ValériePanella, MassimilianoChristaki, EiriniPangrazzi, LucaChristodoulides, MyronPanzner, UrsulaChtourou, HamdiPapadopoli, RosaChu, Chun HungPapadopoulos, GeorgiosChua, Arlène C.Papageorgiou, Ismini E.Chua, Joel V.Papagiannis, DimitriosChuang, Hung-YiPapatsiros, VasileiosChudek, JerzyPappas, Christopher J.Chuev, GennadyPappas, Ioannis S.Chumakov, KonstantinParadkar, PrasadChung, Elaine Yee LinParadowska-Stolarz, AnnaChung, Hee-ChunParan, NirChung, Yoon-SeokPardo-Seco, JacoboCiarambino, TizianaParente, PaolaCiavarella, DomenicoPariani, ElenaCichutek, KlausParisi, SaverioCiftci, Halil I.Parisini, EmilioCiliberti, Maria GiovannaPark, Eun JeongCimolai, NevioPark, Jeong A.Cinar, SuzanPark, Jung-EunCingolani, MarianoPark, WooramCioffi, AndreaPark, Young-JunCirillo, PlinioParodi, AuroraCirillo, StefanoParoli, Maria PiaCitrigno, LuigiParronchi, PaolaCiudad-Blanco, CristinaParvin, RokshanaČižman, MilanPascual, GloriaClarke, SimonPassero, Luiz Felipe DominguesClarke, TainyaPatel, Jay N.Clemens, MarkPatel, PalakCobucci, Ricardo Ney OliveiraPaternostro, FerdinandoCoccia, MarioPathak, RajivCocco, RaffaellaPattanakitsakul, Sa NgaCoe, Christopher L.Paul, Bimal K.Coelho, AdrianoPaulis, GianniCoelho, Luiz Felipe LeomilPavelko, Kevin D.Cofini, VincenzaPawaskar, ManjiriCohen, Elisabeth J.Pedraza Chaverri, JoséCohen, MikaelPeeples, MarkCohen, OferPeiris, DinithiCoia, JohnPellegrinelli, LauraColaprico, CorradoPeltonen, KaritaColasanti, TaniaPeng, HanyongColeman, Anthony WilliamPépin, MichelColeman, ChristopherPerc, MatjazCollins, Natalie D.Perdikogianni, ChrysoulaColomer, Maria AngelsPerdomo, JoseComas-Garcia, AndreuPérez Torres, ArmandoComas-Garcia, MauricioPerez, FranciscoComer, Jason E.Perez, Javier J.Compain, FabricePérez-Castrillón, José LuisConceição-Silva, FátimaPerez-Reche, Francisco J.Condezo Castro, Gabriela NéridaPérez-Rivas, Francisco JavierConners, GregoryPérez-Santos, MartínConstantinescu, Cora M.Pericàs, Juan M.Conte, FerruccioPerrella, AlessandroConti, PioPerumal, VivekanandanContini, CarloPessi, TanjaCoombs, KevinPetersen, EskildCooper, DanPetousis-Harris, HelenCopie, ValeriePetraityte-Burneikiene, RasaCoppeta, LucaPetrakis, IoannisCorbo, DanielePetráš, MarekCordano, ChristianPetsios, KonstantinosCorea, FrancescoPettenuzzo, TommasoCorneau, AurelienPezo, LatoCorral-Gudino, LuisPezzoni, GiuliaCorreia Coelho, Ana CláudiaPflaum, KatherineCosby, LouisePhalipon, ArmelleCosby, Sara LouisePhankitnirundorn, KritCosmi, LorenzoPiano Mortari, EvaCossu, DavidePiantino Ferreira, Antonio JoséCosta, IgorPiazza, Maria FrancescaCosta, LucianaPiccaluga, Pier PaoloCostado Dios, Mari TeresaPichon, MaximeCotelli, MariaPiedra, Pedro A.Coto, EliecerPiekarska, AnnaCrépey, PascalPiepenbrink, Michael S.Criado-Alvarez, Juan JoséPieper, BarbaraCrispo Benedetto, MartinaPierannunzio, DanielaCrocè, Lory SaveriaPieri, MassimoCrocetto, FelicePietrella, DonatellaCrook, BrianPietschnig, JakobCrooke, StephenPignatti, PatriziaCrowley, Andrew R.Pileggi, ClaudiaCruz Hervert, Luis PabloPilotto, José H.Cuesta Martínez, AngelPimenoff, Ville N.Cuesta, AlbertoPimentel, Gustavo DuarteCuestas, María LujánPincus, SethCulebras, EstherPiñeiro, LuisCulqui, Dante R.Pinkas, JarosławCunha, Cristina W.Pinto, DanielaCzarnecki, AdamPion, MarjorieCzerwińska, JoannaPipitone, GiuseppeD’Amelio, RaffaelePires Júnior, Osmindo R.D’Souza, Martin J.Pires, Carla Maria Batista FerreiraDa Rocha Queiroz Lima, Monique R. Q.Pirone, LucianoDa Silva, Henrique BorgesPiroth, LionelDababseh, DeemaPiscopo, MarinaDadar, MaryamPistello, MauroDaguise, VirginiePiubelli, ChiaraDahal, AchyutPivetti, MonicaDahiya, SatinderPizzuti, ClaraDakshanamurthy, SivanesanPlachter, BodoDal Col, JessicaPlans-Rubió, PedroDal Mas, FrancescaPlatteel, Anouk C. M.Dall’Ara, PaolaPlentz, AnnelieDalmastri, ClaudiaPlescia, FulvioDamiani, DanielaPloquin, AurelieDang, ShaonongPniewski, TomaszDangi, TanushreePodhajcer, Osvaldo L.Danieli, Maria GiovannaPolakowski, Nicholas J.Danka Mohammed, Chand ParvezPolašek, OzrenDanovaro-Holliday, CarolinaPollet, JeroenDardas, LatefaPolz-Dacewicz, MałgorzataDarlenski, Razvigor BorislavovPoma, AdolfoDarlix, Jean Luc E.Pomara, CristoforoDarwish, Khaled M.Poniedziałek, BarbaraDas, BasantaPoole, Brian D.Das, BiswajitPoovorawan, YongDaskalakis, EvangelosPorras-Ramirez, AlexandraDavidson, IritPortella, GiuseppeDavies, Neal M.Porter, EdithDavis, Richard E.Portoles, AntonioDavis, William C.Postigo, Idoiade Almeida, Alzira Maria PaivaPostnikov, Eugenede Andrade, Cláudia Regina FurquimPotaczek, Daniel P.de Bree, Godelieve J.Pouliakis, Abrahamde Brito, Rory Cristiane FortesPradenas, EdwardsDe Geest, BartPrado Martins, Rodrigode Graaff, BarbaraPrakas, PetrasDe Jesus, MagdiaPrasad, Ramasare A.de la Torre, Juan C.Prasad, VibhuDe Luca, Paula MelloPratesi, FedericoDe Marco, FedericoPravetoni, Marcode Marzi, Mauricio CesarPrearo, MarinoDe Paula, Sérgio OliveiraPrechl, Józsefde Perio, MariePrehaud, ChristopheDe Rosa, SalvatorePrévost, JérémieDe Sanctis, Juan BautistaPrice, PatriciaDe Sarro, GiovambattistaPrins, Cindy A.De Schrijver, KoenPrintza, NikoletaDe Simone, Salvatore GiovanniPrivitera, Gaetanode Truchis, PierreProcopio, Francesco AndreaDe Vincenzo, RosaProft, ThomasDe Vito, AndreaPrüller, FlorianDe Vito, DanilaPrymula, RomanDe Waure, ChiaraPuerta-Guardo, HenryDe Zotti, MartaPugliese, AndreaDea-Ayuela, María AuxiliadoraPugliese, NicolaDeback, ClairePuig-Barberà, JuanDecaro, NicolaPujol, Flor H.Dechavanne, CéliaPujol, MontserratDecheng, WangPuolakkainen, MirjaDe-Gaspari, Elizabeth NatalPuray-Chavez, Maritza N.Del Fresno Sánchez, CarlosPyo, Hyun MiDel Mistro, AnnarosaQin, KaiDel Riccio, MarcoQin, YueDelgado, RafaelQiu, HuajiDella Polla, GiorgiaQiu, LinDellagostin, OdirQuan, Fu-ShuiDelviks-Frankenberry, KristaQuaye, OsbourneDemarchi, Izabel GalhardoQuinnell, Rupert J.Dénes, AttilaQuintilio, WagnerDeng, TaoRachiotis, GeorgeDeniz, SinanRadcliff, Fiona JaneDepken, CraigRadkowski, MarekDesai, PriteshRadziejewski, CzeslawDesselberger, UlrichRaffard, StéphaneDettori, MarcoRaffetin, AliceDev, SomRagan, IzabelaDey, DebajitRaggi, CarlaDhakal, SantoshRago, LembitDhama, KuldeepRagusa, Maria AlessandraDhawan, ManishRahman, Masmudur M.Dhungyel, OmRahman, Md. TanvirDi Corrado, DonatellaRai, KrishanDi Fazio, NicolaRai, VikrantDi Gennaro, FrancescoRajeevan, Mangalathu S.Di Giamberardino, PaoloRajendran, Ramya LakshmiDi Giovanni, PamelaRajkumar Singh, KalraDi Girolamo, StefanoRajnik, MichaelDi Giuseppe, GabriellaRajput, Mrigendra K. S.Di Martino, GiuseppeRakowski, TomaszDi Micco, SimoneRam, Angia Sriram PradeepDi Nunno, NunzioRamadori, GiulianoDi Profio, FedericaRamalho, Teodorico C.Di Stasi, AntonioRamaprasad, ArkalgudDi Stefano, MariantoniettaRamasamy, RanjanDi Stefano, VincenzoRamirez, Christina M.Di Tucci, ChiaraRamírez-Toloza, GaliaDianatinasab, MostafaRamiro-Cortijo, DavidDias, Lucas D.Ramkissoon, HaywanteeDíaz-Menéndez, MartaRamlo, SusanDickinson, Dale A.Ranieri, JessicaDicks, Matthew D. J.Ranjbar, MaryamDiehl, KatharinaRao, GuodongDielschneider, RebeccaRao, Praveen P. N.Digumarti, RaghunadharaoRapaka, RekhaDigwal, Chander SinghRappoport, NadavDileepan, MythiliRappuoli, RinoDiMeglio, ChloéRascle, PhilippeDini, GuglielmoRashedul Kabir, Arif Md.Dišlers, AndrisRatchina, SvetlanaDixe, Maria Dos AnjosRather, IrfanDobrovolny, Hana M.Rattan, AjitanujDoddapaneni, Bhuvana ShyamRauf, MohdDodds, W. JeanRava, MartaDoerfler, WalterRaveendran, SreejithDoğan, BuhariRavenscroft, NeilDoherty, MarkRawat, KavitaDolan, Brian P.Raza, AliDominoni, MattiaRe, DanielDomnich, AlexanderReal-Fernández, FelicianaDonadu, MatthewReddy, Lebaka Veeranjaneya A.Donohue, WilliamRedican, KerryDonta, SamuelRedlberger-Fritz, MonikaDoosti, AbbasRedwan, Elrashdy M.Dorf, MartinReece, Lisa M.Dorgham, KarimReed, StevenDoritchamou, JustinRehman, HabibDory, DanielRehman, RakhshindaDos Anjos Pires, MariaReichel, Michael P.Dos Santos, Gustavo Rodrigues MakertReid, ScottDouglas, Mark W.Rein, AlanDouxfils, JonathanReiner-Benaim, AnatDowall, StuartRemarque, Edmond J.Dowd, Kimberly A.Resende, TalitaDowling, JenniferRety, StephaneDreesman, JohannesReyes Montes, RocioDreschers, StephanReyes, José LuisDu, XinReyes-del Valle, JorgeDuan, WenmingReyes-Santias, FranciscoDuarte-Escalante, EsperanzaRezza, GiovanniDubaniewicz, AnnaRicci, GiovannaDubey, Jitender PRiccò, MatteoDubey, SaurabhRichards, Stephen J.Duc Hoang, NhatRichter, JanDucatez, MarietteRicke, Darrell O.Duczmal, Luiz H.Rigante, DonatoDuddempudi, Phaneendra K.Rinaldi, SilviaDudhipala, NarendarRíos-Tamayo, RafaelDuharte, Alexander BatistaRipoll, JordiDumache, RalucaRisco-Castillo, VeronicaDundes, LaurenRiva, GiuseppeDuplaga, MariuszRiza, ElenaDuranti, ClaudiaRizza, StefanoDurrani, Aneela ZameerRizzi, ManuelaDustin, LynnRoach, Jared C.Dutta, DebashisRobb, MerlinDwivedi, Gaurav RajRobertson, ShellyDyda, AmalieRobinson, ReneeDzerve, VilnisRoccetti, MarcoEbensperger, GermánRochani, AnkitEcarnot, FionaRockman, StevenEckhart, LeopoldRodrigo, LuisEdwards, Emily S. J.Rodrigues, ArmandaEdwards, Terri G.Rodrigues, Carina De FátimaÉgert, BalázsRodrigues, Celia F.Egyed, LászlóRodríguez Ortega, Manuel JoséEI Ridi, RashikaRodríguez, William R.Eibl, Martha M.Rodriguez-Morales, OliviaEichwald, CatherineRodríguez-Noriega, EduardoEisen, ArriRoehe, PauloEkkehard, BeckRöer, Jan PhilippEksioglu, Erika A.Rogowska, AleksandraEl Bissati, KamalRohaim, MohammedEl Ridi, RashikaRojo, Maria AngelesEl Zahed, SaraRoldão, AntónioElaldi, NazifRolla, SimonaElbestawy, AhmedRomani, AndreaEl-Deeb, WaelRomano, CorradoEldridge, SandyRomano, FerdinandoEl-Duah, PhilipRomano, NiclaEleftheriadis, TheodorosRomer, DanElgendy, MarwaRomero, Carlos H.Elia, DiezRonca, ShannonElit, Laurie M.Roncalli, JeromeEliwa, Mohamed S.Rong, Chan KuanElsayed, Ahmed MostafaRoot-Bernstein, RobertElzey, Bennett D.Rosado, Maria ManuelaEmbregts, CarmenRosano, AldoEndo, MakotoRosario-Cruz, RodrigoEpelde, FranciscoRoselle, Gary A.Erdman, Dean D.Rosenthal, KenEscaffre, OlivierRossi, Noreen F.Escors, DavidRossi, OmarEscudero-Pérez, BeatrizRossiter, PaulEsposito, MassimilianoRosso, AnnalisaEstevez-Diz, Maria Del PilarRotte, AnandEsworthy, Robert StevenRoubalova, KaterinaEtiler, NilayRoukens, Anna Helena ElvireEufemi, MargheritaRovin, Richard A.Evans, Thomas GeorgeRovo, AliciaEverett, Helen E.Rowlands, David J.Exbrayat, Jean-MarieRozek, WojciechEyase, FredRuckwardt, Tracy J.Fabbrini, PaoloRuggieri, AnnaFaburay, BontoRuggiero, CarmelinaFagone, PaoloRuisoto, PabloFaheem, MuhammadRuiz-Argüelles, GuillermoFakhoury, HanaRupert, AbeleFal, AndrzejRus, Horea G.Falfan-Valencia, RamcesRusso, GiuliaFalkinham, Joseph O.Russo, MomtchiloFallarini, SilviaRustam, FurqanFalzone, LucaRusticeanu, MonicaFan, HuiyingRuță, SimonaFan, ShufangRwegerera, Godfrey M.Fang, Chee-MunRybka, JakubFantini, Maria PiaRybtsov, StanislavFaquim-Mauro, Eliana L.Rychtar, JanFara, Gaetano MariaRyffel, BernhardFarina, ClaudioRyu, Mun-HoFaron, Matthew L.Saad, Mohd ZamriFarone, Mary B.Saade, ElieFarooq, MuhammadSaba, AmalFarronato, MarcoSabbah, DimaFarrukee, RubaiyeaSabbatucci, MichelaFarsang, AttilaSabino-Silva, RobinsonFattore, LianaSabir, Ahmad JawadFehl, AmySabourin, CarolFeito, JorgeSacchini, FlavioFeldman, CharlesSacco, PierFeng, NingguoSack, UlrichFeng, ZongdiSadaoka, TomohikoFeola, AlessandroSadraeian, MohammadFernandes, Maureen H. V.Saggu, GagandeepFernández, Juan JoséSahm, Laura JaneFernández, SoledadSahu, Sanjaya KumarFernando, FerreiraSaid, Elias A.Ferrantelli, FlaviaSaid, MohamedFerrara, FrancescoSaito, ReikoFerrara, PietroSaito, YukiFerraz, Maria PiaSajid, MalikFerreira, FernandoSakka, VissariaFevang, BørreSalamon, DominikaFichtenbaum, Carl J.Salas, SofiaFigueras, AlbertSalazar, FabíanFilipek, AgnieszkaSalehi, MohammadrezaFilippini, FrancescoSalerno, LauraFilmann, NatalieSallam, MalikFine, PaulSalman, MoFinelli, CarmineSalmanton-García, JonFinsterer, JosefSaltoglu, NeseFionda, BrunoSalzberger, BerndFiore, MariaSamal, SangramFirek, AnthonySambri, VittorioFischer, FlorianSamji, Naga SwethaFittler, AndrásSampaio, RuteFlisiak, RobertSam-Yellowe, TobiliFoddis, RudySánchez Ojeda, María AngustiasFoglia, EfremSanchez Santos, ManuelFol, MarekSanchez, David JesseFolgori, AntonellaSancineto, LucaFonseca, Gregory J.Sander, ValeriaFonseca, KevinSandhu, KiranFonseca, MagileSandonà, DoriannaFontana, StefanoSandulescu, OanaFonzo, MarcoSanftenberg, LindaFoppa, IvoSanjay, SrinivasanFormanowicz, PiotrSantacroce, LuigiForoncewicz, BartoszŠantak, MajaFox, JulieSantana, Andrés GonzálezFraga, HugoSantini, AntonelloFraile, LorenzoSantoro, Michele CosimoFraile-Martinez, OscarSapp, MartinFraleoni-Morgera, AlessandroSarapultsev, AlexeyFranco, DavidSardu, CelestinoFranzoni, GiuliaSardu, ClaudiaFrassanito, AntonellaSaribas, SuatFrati, PaolaSarigiannis, DimosthenisFratini, FilippoSarkar, AmritaFreedman, AndrewSarkar, SubenduFreer, GiuliaSarno, LauraFregeneda-Grandes, Juan-MiguelSarría Santamera, AntonioFreiberg, Alexander N.Sato, HiroshiFreire-de-Lima, Celio GeraldoSato, RyokoFrey, KurtSattler, David N.Frickmann, HagenSauleda, SilviaFrietze, KathrynSavellin, Gianni GoriFröhlich, EleonoreSaviano, AngelaFrossard, Jean-PierreSavinelli, StefanoFthenakis, GeorgeSaxena, PoojaFu, JianingSaxena, ShrishtiFu, QinSayed, Ibrahim M.Fuchs, LiorScaramuzza, AndreaFuentes Quinteros, EduardoSchaefer, Brian C.Fujisawa, TakaoSchäffer, Alejandro A.Fukasawa, TakemichiSchechtman, DeborahFukumoto, YoshihiroSchepens, BertFuller, FredSchieck, EliseFuller, Frederick J.Schirtzinger, Erin ElizabethFurer, VictoriaSchmid, StephanFusco, RobertaSchmidt, BarbaraFux, Christophe AndreasSchnierle, Barbara S.Gacesa, RankoSchoenhagen, PaulGadd, TuijaSchrama, DeniseGagner, Jean-PierreSchreiner, SabrinaGaikwad, HanmantSchroeder, Harry W., Jr.Gairola, SunilSchwermer, HeinzpeterGajdács, MárióScopetti, MatteoGala, Rikhav P.Scott, DavidGalanis, AlexisScott, PeterGalanis, PetrosScotti, ClaudiaGalante, DomenicoSebastiani, GiovanniGałas, AleksanderSee, Kay ChoongGalindo-Cardiel, Iván JoséSeed, SheilaGallè, FrancescaSegala, FrancescoGallelli, LucaSeidl, MaximilianGalleri, GraziaSeike, MasahiroGambardella, ClaudioSelim, Abdelfattah M.Gambaryan, AlexandraSellner, JohannGambino, Caterina MariaSelva, Kevin J.Gameiro, SteveSelvaraj, PeriasamyGammeren, Adriaan J. VanSelvey, LindaGan, SamuelSeminari, ElenaGan, Samuel Ken-EnSemino, ElenaGanesan, KumarSeng, Jun Jie BenjaminGao, YuanŠenigl, FilipGarcía Iglesias, María JoséSeo, Ho SeongGarcía-Cózar, FranciscoSeo, Keun-SeokGarcía-Dorival, IsabelSeo, Sang HeuiGarcin, DominiqueSepand, Mohammad RezaGarfield, SaraSergi, ConsolatoGarner-Spitzer, ErikaSerrano, AntonioGarnes, Natalie DaileySerrano, Dolores R.Garofalo, CinziaSerša, GregorGarofalo, Roberto P.Servidio, RoccoGarofolo, GiulianoSessa, FrancescoGarry, RobertSethi, GautamGarushyants, Sofya K.SeyedAlinaghi, SeyedAhmadGarvy, Beth A.Shah, GunjanGasperini, BeatriceShah, RasoolGasque, PhilippeShakya, AkhileshGatto, FrancescaShams, Abdullah BinGautam, AvneeshShan, SonghuaGavrielatou, NikiShan, Xiaojun (Gene)Gazarini, Marcos LeoniShankar, Esaki MuthuGe, BaoxueShao, WeiGe, WenzhenShapiro, Stuart Z.Geary, SeanSharma, AdityaGeng, QibinSharma, Ashish RanjanGenovesi-Ebert, FedericaSharma, AtulGentile, DeborahSharma, MeghaGentile, StephanieSharma, SavitriGeorgakopoulou, EleniSharma, Shalini K.George, SantoshSharma, Tasneem P.Georgescu, AdrianaSheek-Hussein, MohamudGerhard, MarkusShehata, Awad A.Gerlich, WolframShekhar, RahulGermain, Ronald N.Sheng, WeiGeronikolou, StylianiSherman, Amy C.Gershburg, EdwardSherry, LeeGershon, Anne A.Sherwani, Mohammad AsifGerymski, RafałShestopalov, Alexander M.Gessler, FlorianShi, JishuGharbi, JawharShi, MiaomiaoGhayda, Ramy AbouShields, LindseyGhibaudo, GiovanniShima, YoshihitoGholizadeh, PouryaShimada, MasaruGhosh, MrinmoyShimizu, HiroyukiGhosh, PrithwiShimizu, JunichiGhosh, SomiranjanShimizu, TakeyukiGhosh, SujayShimizu, TomokazuGiacomelli, MauroShimodaira, ShigetakaGiambra, VincenzoShimotai, YoshitakaGianfredi, VincenzaShirai, JyunsukeGiangaspero, MassimoShirai, TsuyoshiGiannetto, ClaudiaShirato, KazuyaGiavedoni, PriscilaShittu, IsmailaGidlund, MagnusShivanna, VinayGil De Miguel, ÁngelSholukh, AntonGil, Eric S.Shrestha, BinitaGilbert, LeonaShridhar, SurekhaGilbert, Peter B.Shringi, SmritiGillinder, KevinShrivastava, Saurabh RamBihariLalGillot, ConstantShtro, AnnaGilmour, StuartShults, Elvira E.Gimbel, SteveSi, FushengGiordano, RicardoSi, LeiGiorgi, ElenaSiau, Ching SinGirard, MarcSiddiqui, ArifGiraudo, Maria TeresaSignore, FabrizioGirault, GuillaumeSignori, AlessioGirija, RamakrishnanSikalidis, AngelosGirrbach, Felix F.Sikora, MariuszGiudice, ElisabettaSilva, InêsGiurisato, EmanueleSilva, Marcelo SousaGlebov, Oleg O.Silva-Caso, Wilmer GianfrancoGnat, SebastianSilva-Costa, CatarinaGodman, BrianSilva-Júnior, AbelardoGodoy, PereSilvestrini, LuciaGoel, AmitSimbirtsev, AndreyGöhring, LutzSimecka, Jerry W.Gołębiewska, JustynaŠimić, IvanaGolender, NataliaSimons, GregoryGomez Del Moral, ManuelSimpson, MareeGómez, Carlos HernandoSinganalur, Nagendrakumar BalasubramanianGomez, Carmen ElenaSingh, Amarinder SinghGómez-Dantés, HéctorSingh, AmbrishGonçalves, LídiaSingh, Amit K.Goncalves, VivianeSingh, BhagirathGonçalves-Carneiro, DanielSingh, Brajesh KumarGonçalves-Pereira, JoãoSingh, Shailendra PratapGonciarz, WeronikaSingh, ShaktiGong, MinghaoSingh, Sunil KumarGonzález Cappa, Stella MarisSingh, VineetGonzalez Domenech, Carmen MariaSingla, RajeevGonzález, Florenci V.Sinha, DiptoGonzález, María EugeniaSinigaglia, AlessandroGonzalez–Curiel, IrmaSinopoli, AlessandraGonzález-Domínguez, IreneSionov, RonitGonzález-Granado, Luis IgnacioSipos, FerencGonzález-López, Tomás JoséSipsas, Nikolaos V.Gonzalez-Parra, GilbertoSiristatidis, CharalamposGonzález-Rodríguez, AlexandreSivaraman, VijayGonzález-Scarano, FranciscoSkwarczynski, MariuszGoodman, Alan G.Sloan, ChantelGoodrich, Raymond P.Smaoui, SlimGorczynski, ReginaldSmith, KerryGori, DavideSmith, Rex N.Gorini, FrancescaSmith, Stephen A.Goto, AyaSnell, Christopher R.Gouandjika-Vasilache, IonelaSnowden, JessicaGourapura, RenukaradhyaSoares, HelenaGowans, EricSobieszczańska, MałgorzataGożdzik, KatarzynaSochocka, MartaGraciotti, MicheleSoe, Zar ChiGranato, MarisaSoentjens, PatrickGrand, RogerSogawa, NorioGranito, AlessandroSohrabi, YahyaGrant, MichaelSokolov, AlexeyGray, BenSoldan, Samantha S.Gray, WayneSoliman, MohamedGraziano, ElenaSolimando, Antonio GiovanniGreim, HelmutSolopov, PavelGrieco, TeresaSoloveva, VeronicaGrigorov, BoyanSominskaya, IrinaGroff, JosephSomu, PrathapGroome, Michelle J.Son, Young-OkGroppelli, ElisabettaSonderup, Mark W.Gruters, Rob A.Song, WenxiaGrzegorczyk, IzabelaSonnino, GiorgioGrześk, GrzegorzSoriano-Sarabia, NataliaGu, ChenjianSotelo, JulioGu, ChunyangSoteriades, ElpidoforosGuan, MinhuiSousa, Ivanildo P.Gudey, Shyam KumarSousa, Sílvia A.Guérin, Diego M. A.Soyfoo, MuhammadGueron, GeraldineSpataro, PietroGuerra, JessicaSpearman, PaulGuerriero, VincenzoSpinelli, Francesca RomanaGuest, Johnathan D.Spiro, David J.Guglielmi, GiovanniSpitzer, MartinGuilherme, LuizaSpreat, ScottGuillaume, Thierry A.Sridhar, SiddharthGuillen-Grima, FranciscoSrikiatkhachorn, AnonGujski, MariuszSrivastava, YogeshGulla, KrishnaSt. Cyr, John Albert Gulve, NitishStacchini, LorenzoGuo, HuichenStalika, EvangeliaGuo, XizhiStamovlasis, DimitriosGuo, Zong ShengStanek, AgataGyӧrke, AdrianaStanton, Sasha E.Ha, DatStavropoulou, ElisavetHaag, FriedrichStear, MichaelHaataja, SauliSteele, DuncanHaddock, Christopher KeithSteele, Edward J.Hägerling, RenéStefanizzi, PasqualeHai, RongStefanoff, PawelHalassy, BeataStefanska, AnnaHalicioglu, FerdaSteger, GertrudHallam, Jeffrey S.Steinborn, MichaelHalle, StephanStendahl, OlleHaller, BernhardStephens, EdwardHalperin, Daniel T.Sternberg, RobertHalperin, John J.Stevanović, VladimirHalpern, MichaelSticherling, MichaelHaluza, DanielaStorsaeter, JannHamasaki, YoshifumiSt-Pierre, YvesHamilton-West, ChristopherStreatfield, Stephen J.Hammerl, Jens AndréStreck, André FelipeHammerstad, Sara SalehiStrutt, Tara M.Hampson, IanStrzemski, MaciejHampson, LynneSu, ErikHan, JinmingSu, ShanHancu, GabrielSu, ZhaohuiHand, Ivan L.Subbiah, MuruganHang, JunSubbian, SelvakumarHanif, ShumailaSubhan, Md AbdusHann, Hie-WonSubudhi, SonuHantz, SébactienSuda, YasuoHappel, ChristineSudarikov, AndreyHara, MegumiSuffritti, ChiaraHarald, MatthesSuganuma, KeisukeHarapan, HarapanSuggs, SuzanneHarashima, NanaeSugiyama, ToruHarkiss, GordonSuhrbier, AndreasHarper, DianeSui, YongjunHarrer, ThomasSullivan, Erin E.Harris, Audray K.Sullivan, John StephenHarris, BerniceSummerfield, ArturHaruna, TakenoriSun, BaoqingHarvey, BartSun, CaijunHasegawa, MakotoSun, ChenyuHashimoto, MakotoSun, HongleiHashizume, HideoSun, XiangjieHaslwanter, DeniseSun, ZhaoxiHassan, Hossam M.Sun, ZhichenHassan, MohamedSundström, Mare LõhmusHassan, Sherif T.S.Sung, Ji-YounHassandarvish, PouyaSunwoo, Sun-YoungHässig, MichaelSurachetpong, WinHathout, RaniaSurapaneni, Krishna MohanHatmal, Ma’mon M.Suresh, SnehaHato, TaiSurve, Chinmay R.Hattori, ToshioSuryadevara, ManikaHatzakis, AngelosSuryadevara, NaveenchandraHauptmann, SteffenSuryawanshi, RahulHausdorff, William P.Suschak, John J.Hawkes, DavidSuter, MarkHayashi, TomokoSuzuki, RyosukeHayes, Sidney J.Suzuki, TakashiHe, ChunboSuzutani, TatsuoHe, DaihaiSvoboda, MartinHe, QiushuiSzamotulska, KatarzynaHearing, PatrickSzczepanek, JoannaHecht, MichaelSzczepańska, MariaHedges, JodiSzilák, LászlóHedman, KlausSzmyd, BartoszHefferon, Kathleen L.Szollosi, AndrasHegazy, WaelSzymborski, TomaszHeger, LukasTada, TakuyaHeldal, KristianTafuri, SilvioHeller, DanTahamtan, AlirezaHelsloot, IraTaheri- Ledari, RezaHelton, Rebekah R.Takahashi, TakayukiHemmati, ShivaTakahashi-Omoe, HiromiHemphill, AndrewTakam Kamga, PaulHengartner, NicolasTakamura-Enya, TakejiHenics, TamásTakeno, MitsuhiroHentzien, MaximeTakita, MorihitoHerdeiro, Maria TeresaTalaga, PhilippeHernandez, JesusTalarek, EwaHerold, BetsyTalebi Bazmin Abadi, AminHerrera, GuadalupeTalias, Michael A.Herzum, AstridTamam, LutHess, KarlTamayo, EstherHicham, ElmsellemTan, Andy Hee-MengHIjikata, AtsushiTan, Bee KangHindmarsh, DianeTan, MingHinkula, JormaTan, Tina Q.Hirsch, IvanTanabe, ShihoriHo, Jar-YiTanaka, JunkoHoang, Van ThuanTanaka, MasakazuHoefnagel, MarcelTandlich, RomanHoepner, RobertTandon, RiteshHoffmann, BerndTaneja, NeelamHoffmann, MarkusTang, JulianHogenEsch, HarmTanislav, ChristianHogue, Brenda G.Taniyama, DaikiHomma, AkiraTantak, MukundHomonnay, ZalánTardif, VirginieHonda-Okubo, YoshikazuTardivo, StefanoHong, Bo-YoungTarighi, SaeedHong, Chang YuTarr, Philip E.Hong, HuixianTashima, ToshihikoHong, MyungheeTassaneetrithep, BoonratnHooper, Douglas C.Tasso, AlessandraHorii, ToshihiroTatsumoto, MunetoHorstman, KlasienTaus, NaomiHorta, Marco Aurélio PereiraTavares, PedroHošnjak, LeaTavassoly, ImanHossain, MohammedTaylor, MatthewHostiuc, MihaelaTazzari, MarcellaHouen, GunnarTe Velthuis, Aartjan J. W.Houhamdi, LindaTeljeur, ConorHowe, Daniel K.Telli, AsliHowell, Mark C.Temchura, VladimirHoyne, Gerard FTemperton, NigelHozbor, DanielaTempesta, MariaHrdy, JiriTemsah, Mohamad-HaniHsia, Shih-MinTennstedt, PierreHsiao, Hui-HuaTerlizzi, MichelaHu, JiafenTernák, GáborHu, MinTerzian, Ana C. B.Hu, YangTesoro, EmilianoHuang, ChangTesta, AnnaHuang, Jason C.Thackray, AlanaHuang, JiachenThai, Khac-MinhHuang, Sheng-WenThaker, Youg RajHuang, Yi-Hui ChristineThepparit, ChutimaHuang, ZiliangTheron, JacquesHuber, VictorThielen, Beth K.Huet, EricThiruvengadam, MuthuHuhtaniemi, IlpoThomas, PaulineHulsen, TimThomas, Pauline A.Hurst, Tara PatriciaThomas, Roger E.Hussain, SaadThomas, WayneHuxford, TomThompson, AndrewHwa, Kuo-YuanThuan, Hoang VanHyder, Abd-AllahThwaites, LouiseIannario, MariaTimofeeva, Angelika V.Iannetta, MarcoTimofeeva, Tatyana A.Ibrahim, AbdelhameedTinelli, AndreaIdda, Maria LauraTiriveedhi, VenkataswarupIdoate, MiguelTiscar, Pietro G.Idris, OlayinkaTo, Wai MingIhle, AndreasTokiya, MikikoIijima, NoriakiTolbert, William DavidIlic, IrenaToledano-Magaña, YanisIllario, MaddalenaToledo, Karina A.Ilyinskii, Petr O.Tomar, VartikaImashuku, ShinsakuTomaszewska, EwaImhoff, RolandTondo, GiacomoImmovilli, PaoloTonetti, LorenzoImran, MohdToniolo, AntonioImran, MuhammadTorán-Monserrat, PereInagaki, YoshinoriToranzo, Alicia E.Ince, Ikbal AgahTormanen, KatieInchingolo, FrancescoTorner, NuriaInfante, MarcoToro, LuisInfantino, MariaTorras, JuanInokuchi, RyotaTorres-Fernández, OrlandoIorio, Ronald M.Toth, EricIovino, FedericoToubai, TomomiIrigoyen-Camacho, María EstherToyos, Juan R. De LosIrobi, JoyTrabaud, Mary-AnneIsakova-Sivak, IrinaTrabelsi, KhaledIsegawa, YujiTrefry, John C.Ishigaki, HirohitoTremolada, MartaIshigaki, YasuhitoTretyn, AndrzejIshikawa, TomohiroTrevisan, AndreaIslam, Md RabiulTricarico, DomenicoIsmael, M. IsabelTrinh, Kieu The LoanIsmail, Ashrafali MohamedTrinité, BenjaminIsoda, NorikazuTripathi, Satyendra C.Issagouliantis, MariaTrombetta, CarloIstıflı, Erman SalihTruong, ThangIto, EtsuroTsai, Wen-HsienIvanova, Krasimira BorislavovaTsakogiannis, DimitrisIvanova, Olga E.Tsay, Shwu-ChenIzopet, JacquesTschernig, ThomasJacobson, MireilleTselebis, AthanasiosJadhao, Samadhan J.Tsetsos, NikolaosJain, ShilpiTsilibary, Effie-Photini C.Jain, SwatiTsubaki, MotonariJaiswal, Rishi K.Tu, Hung-PinJaiswal, SwatiTuells, JoséJakovljevic, MihajloTuller, TamirJalah, RashmiTun, Mya Myat NgweJalava, KatriTung, Ho-JuiJalili, AhmadTung, Tao-HsinJames, JoeTurano, PaolaJancloes, MichelTurbow, DavidJankovic, MomciloTurco, JeniferJankowski, MateuszTurillazzi, EmanuelaJansen Van Vuren, PetrusTurksen, KursadJansen, Christine A.Tussardi, Ilaria ToccoJanuszkiewicz-Lewandowska, DanutaTutukina, Maria NikolaevnaJaskuła, EmiliaTvrda, EvaJeannot, EmilienTye, Gee JunJena, Ravindra KumarTylicki, LeszekJenkin, DanielTyrkalska, SylwiaJenner, Dominic C.Ucciferri, ClaudioJentarra, Garilyn M.Uchida, ShizukaJeong, HanseobUchiyama, SusumuJeong, SeogsongUddin, Md. BashirJeong, SeriUema, MasashiJerkic, MirjanaUgalde, Juan E.Jerse, Ann E.Ulasov, IlyaJeulin, HélèneUlu, ArzuJha, Rajiv KumarUmer, MuhammadJiang, JiekeUnajak, SasimanasJiang, YifengUnluturk, UgurJiantai Timothy, QiuUrbaniak, MonikaJin, AndrewUsui, TatsufumiJin, HongpingUzelac, AleksandraJin, YongVagni, MoniaJohnson, Dylan M.Vaismoradi, MojtabaJohnson, Mark S.Vajo, ZoltanJohnston, Michael J. W.Valasoulis, GeorgeJonas, Kai J.Valencic, EricaJones, ChrisValenzuela, RodrigoJones, IanValkenburg, Sophie A.Jones, Kathryn MarieVallée, AlexandreJones, MarkValls Martínez, María Del CarmenJones, Rodney P.Van Der Pol, Leo A.Jongkaewwattana, AnanVan Der Sande, MarianneJosé Luis, BarrancoVan Der Zeijst, B.A.M. (Ben)Jose, MarcoVan Dijk, AlbieJoshi, GauravVan Dijk, Jitse P.Juarez, RubenVan Domselaar, RobertJulkunen, IlkkaVan Gorp, Eric C. M.Junaidi, JunaidiVan Krieken, RichardJung, Sung-JuVan Santen, VickyJung, Tae SungVanham, GuidoJuris, JansonsVann, WillieJurisic, VladimirVaradwaj, PradeepKabamba Nzaji, MichelVarani, LucaKabir, K. M. ArifulVardasca, RicardoKabir, RussellVargas, Alessandro N.Kafarski, PawełVargas, SergioKahar, PrihardiVarju, CeciliaKajiyama, HiroshiVartholomatos, GeorgeKalachev, Leonid V.Vasile, FrancescaKalam, Md. AbulVasquez, IgnacioKaliamurthi, SatyavaniVaughan, AshleyKalichman, SethVavvas, Demetrios G.Kalkum, MarkusVázquez-Domínguez, EvaristoKałucka, Sylwia KatarzynaVega, BernardoKamachi, KazunariVelander, William H.Kamensek, UrskaVelasco-Villa, AndrésKamigaki, TaroVelázquez-Juárez, GilbertoKaminski, Robert W.Velazquez-Salinas, LauroKammerer, RobertVenancio, Emerson JoséKanada, MasamitsuVenkatachalapathy, MuthukumaranKanai, YutaVenkatesan, GnanavelKanatani, YasuhiroVenkatesan, PradhibKanda, TatsuoVenkatraman, SitalakshmiKandimalla, RameshVentura, FrancescoKantar, AhmadVergadi, EleniKao, Chai-LinVerheul, Marije K.Kao, Shung-TeVerhoeven, AdrieKapetanakis, EmmanouilVerjans, Georges M. G. M.Kaplan, Robert M.Verma, MukeshKapuria, DevikaVerma, ShailendraKarampour, SajadVerros, George D.Karanika, StylianiVersacci, PaoloKariminik, AshrafVersteeg, LeroyKarlsson Hedestam, Gunilla B.Vervoort, LeentjeKarlsson, ErikVesikari, Timo H.Karp, Jonathan D.Vetrugno, GiuseppeKarpova, Olga V.Vicente, Giner GalvánKashiwagi, SatoshiVictor, Jefferson RussoKashyap, VivekVictorio, Carla Bianca LuenaKaufman, David A.Viganò, MauroKaufusi, Pakieli H.Vignier, NicolasKaur, HargobinderVilella, AnnaKaushik, SamanderVilibić-Čavlek, TatjanaKausik, DattaVilla, LucaKavaliauskiene, SimonaVillalba-Montoro, RafaelKawamura, YoshikiVillarruel-López, AngélicaKawsar, Sarkar M. AbeVillela-Nogueira, CristianeKe, HanzhongVimercati, AntonellaKeeler, ShamusViranaicken, WildrissKeikha, MojtabaVirtaneva, KimmoKelly, GabrielleVisvizi, AnnaKelly, Kevin M.Visweswaran, Ganesh RamKempster, Sarah L.Vita, RandiKenney, Joan L.Volpert, VitalyKenry, KenryVon Eggeling, FerdinandKenzaka, TsuneakiVon Hunolstein, ChristinaKerdsin, AnusakVon Kries, RüdigerKesari, Kavindra KumarVon Laer, DorotheeKesherwani, Varun KumarVuillefroy De Silly, RomainKęsik-Brodacka, MałgorzataVujaklija Brajković, AnaKeswani, TarunVuković, VladimirKezuka, TakeshiVuong, Quan-HoangKhafizov, Kamil F.Vyatkina, Kira V.Khajanchi, SubhasWada, HideoKhalid, HattafWaghray, AvinashKhalil, AtefWagner, Gert G.Khalil, IbrahimWagner, LudwigKhalique, ChaudryWaisberg, JaquesKhan, AbbasWakiguchi, HiroyukiKhan, AsifWalduck, Anna K.Khan, BilalWallace, ManolisKhan, Owais A.Wallon, MartineKhan, RehanWalwyn, Wendy M.Khan, Shah AlamWan, Thomas T. H.Khan, Yusra HabibWan, YanminKhatua, Atanu K.Wang, BeiKhlebnikov, Andrei I.Wang, BoKhor, Seik-SoonWang, Chi-ChuanKhubchandani, JagdishWang, CynthiaKiazyk, SandraWang, Fun-InKido, YasutoshiWang, HaoqingKiiamov, AiratWang, HongningKijewska, MonikaWang, Hurng-YiKim, BeomjoonWang, Jee-ChingKim, Hae YoungWang, JiKim, Hei-sungWang, JiaweiKim, HoonWang, JiufengKim, Hwa-JungWang, JunmeiKim, Hyun SooWang, Li-ChiehKim, Hyun-oukWang, LihuaKim, In-JeongWang, MinghangKim, JayoungWang, PengfeiKim, Jin HeeWang, Robert Y.-L.Kim, Ryong NamWang, WeiKim, SangjuneWang, WenjunKim, SeilWang, William Yu ChungKim, SungwonWang, XianwenKim, SungwookWang, YeKim, TarkWang, YuhangKim, Young KookWang, ZhenKirbiš, AndrejWang, ZongjieKircheis, RalfWann, Lee SamüelKirchenbaum, Greg A.Want, Muzamil YaqubKirk, Natalie M.Ward, Lucy AnnKirman, JoannaWasti, Sharada P.Kiseleva, IrinaWatt, SimonKishimoto, JiroWattegedera, SeanKiszewski, TonyWaye, Mary M. Y.Kita, ToshihiroWebb, FernKitagawa, SeiichiWeber, Georg F.Kitamura, KouichiWebley, WilmoreKiulia, Nicholas M.Weger, StefanKiyohara, TomokoWei, FeixueKlafack, SandroWei, HongpingKleber, MartinaWei, JinKlegerman, Melvin E.Wei, JinhongKlimowicz-Bodys, Małgorzata D.Weisblum, YiskaKnapp, PeterWeiss, ChristelKnittler, Michael R.Wertz, Philip W.Kobayashi, TetsuroWesthoff, Mike-AndrewKobeissy, FirasWiedermann, WolfgangKocazeybek, BekirWieland, AndreasKohara, MichinoriWilkinson, EwanKohler, James J.Willcox, MarkKokudo, TakashiWilliamson, Ethel DianeKolbe, MichaelWilm, MatthiasKoleilat, IssamWilson, Alphus D.Kolev, MikhailWilson, Douglas R.Kollias, AnastasiosWilson, WilliamKomase, KatsuhiroWitczak, Zbigniew J.Koncz, GáborWitkowski, WojciechKondo, HidehiroWolfe, DanielKondoh, TakeshiWölfel, RomanKonstantinidis, Theocharis G.Wolfraim, LarryKorfitis, ChrysovalantisWong, Chi-MingKornya, LaszloWong, Fai-ChuKorompelis, PorfyriosWood, Kelly E.Korona-Glowniak, IzabelaWoods, Charles R.Korva, MisaWoods, Jon P.Kościelska-Kasprzak, KatarzynaWoods, Marion L.Kosik, IvanWozniak, JacekKoskan, AlexisWozniak-Knopp, GordanaKosmas, IoannisWoźniakowski, GrzegorzKostantinidis, TheocharisWu, Jack H. Z.Kou, GangWu, JianxuanKounis, NicholasWu, Jun-JunKouokam, Joseph CalvinWu, Jyh-JengKowalska, JustynaWu, YahongKozlov, AndreyWu, ZhuoxunKozlovskaya, LiubovWünsch, DésiréeKrähenbühl, StephanWurm, Florian M.Králik, RomanXavier, Sastre-GarauKratzer, BernhardXi, JinxiangKrause, FrankXia, MingKreitmann, LouisXiao, YongliKrejbich-Trotot, PascaleXoconostle-Cázares, BeatrizKreutzberger, Alex J. B.Xu, Jian (Japan)Kreyenberg, HermannXu, Jian (USA)Krishna, VenkatramanaXu, KaiKrishnakumar, DevadasXu, YongKrishnamoorthy, ParamanandhamYabal, MonicaKrishnan, VengadesanYadav, Dharmendra KumarKrishnasamy, GopinathYadavalli, TejabhiramKristian, PavolYager, EricKroeker, AndreaYakobson, Boris A.Kropp, MartinaYamanaka, AtsushiKrug, PeterYamout, Bassem I.Krumbholz, AndiYan, ZhipengKrystal, MarkYang, GuangzeKrzysztoń-Russjan, JolantaYang, HaiyanKrzyżowska, MałgorzataYang, HangKüchenhoff, HelmutYang, KunKues, John R.Yang, LiKuhlmann, F. MatthewYang, LiminKujan, OmarYang, MingKumagai, MakikoYang, MingchongKumar, AjayYang, Ming-HuiKumar, AmitYang, Nan-PingKumar, AnandYang, TiantianKumar, ManojYang, Wei-hsiungKumar, PraveenYang, XinKumar, RajYang, YichiKumar, RavinderYang, YunchenKumar, RiteshYang, ZhiKumar, RobinYao, XiaoJianKumar, SunilYap, Sook FanKumararatne, DinkanthaYaparla, AmulyaKumaraswamy Naidu, ChitralaYawn, BarbaraKunze, MichaelYe, GangKurakula, MalleshYe, ZhongfengKurup, DrishyaYeh, Ta-ChuanKus, KrzysztofYeh, Tsu-MingKusaka, MamoruYehuda, ShoenfeldKusuma, Yadlapalli S.Yellapu, Nanda KumarKuter, Barbara J.Yen, Cheng-FangKuzmina, Natalia A.Yeoh, Eng-kiongKwiatek, AgnieszkaYeremeev, VladimirKwok, Henry Hang FaiYeung, Andy Wai KanKwon, Hyuk-JoonYilmaz, SezaiKwon, OkyuYin, LiKyriakoulis, Konstantinos G.Yip, JoanneLa Torre, GiuseppeYoda, TakeshiLabuschagne, KarienYon, Dong KeonLabuz-Roszak, BeataYong, LeijianLaforenza, UmbertoYoo, Han SangLager, MalinYoon, HachungLahariya, ChandrakantYoon, WonsuckLai, AlexanderYorsaeng, RitthideachLai, Chih-ChengYoshida, Jun’ichiLai, Daniel W. L.Yoshida, NoriakiLake, Douglas F.Yoshinaga, JunLakshmanappa, Yashavanth ShaanYou, MinliLalsiamthara, JonathanYoun, Bo-YoungLand, Kenneth C.Younes, HusamLandires, IvanYounes, ZaidLang, FengchaoYoung, Howard A.Lange, AndrzejYura, YoshiakiLanghorst, Bradley W.Yusriadi, YusriadiLantieri, FrancescaYuta, HikichiLarabee, Jason L.Zablackis, EarlLaranjeira, CarlosZafar, MuhammadLarrous, FlorenceZafer, NajimLarsen, Carsten SchadeZaffina, SalvatoreLarsen, DavidZagożdżon, RadosławLarson, PeterZahid, Muhammad N.Larue, Ross C.Zając, ZbigniewLasrado, NinaadZakaryan, HovakimLatini, AlessandraZamperin, GianpieroLatkin, CarlZanella, IsabellaLau, Ho Yin EricZannella, CarlaLau, Joseph Tak-faiZaramella, PatriziaLaumaea, Annemarie E.Zárate, SeleneLaunay, OdileZaravinos, ApostolosLaurent, EmelineZarębska-Michaluk, DorotaLaurenti, PatriziaZarobkiewicz, MichałLaurie, KarenZavala-Alonso, Norma-VerónicaLavender, KerryZavras, DimitrisLavoie, Kim L.Zefferino, RobertoLaw, John Lok ManZeichner, Steven L.Lazaroiu, GeorgeZelek-Molik, AgnieszkaLe Doaré, KirstyZeleny, ReinhardLe Pendu, JacquesZell, RolandLe, Hoang-ThanhZeng, WeiguangLeal, Ana MendesZenk, JohannesLeclerc, DenisZenteno-Galindo, Arturo EdgarLecureux, JonathanZé-Zé, LíbiaLedda, CaterinaZhang, ChengxinLeder, KarinZhang, Daniel XinLee, Cindy H. S.Zhang, DingmeiLee, Jae-HoZhang, GongLee, Jai-WeiZhang, GongyiLee, JihyeZhang, HeLee, Joon KeeZhang, JinyangLee, Keun HwaZhang, LianjunLee, Kyung-YilZhang, LiboLee, MinwooZhang, LongLee, Tzong-ShyuanZhang, PengfeiLee, Wei-ChiehZhang, ShengLee, Wen-SenZhang, ShixiongLee, Yuan-TiZhang, TianxiaoLefebvre, FrancoisZhang, XiaorongLegaz, IsabelZhang, XiaotingLegler, PatriciaZhang, XinxingLehtonen, LasseZhang, YibingLeibovitz, Eugene L.Zhang, YingLeitão, JoãoZhang, YuexiuLeitão, Jorge H.Zhang, YuqingLeitner, Wolfgang WZhao, FangzhuLekcharoensuk, PorntippaZhao, KaiLelešius, RaimundasZhao, QinLemmermann, NielsZhao, QuanyiLeno-Durán, EsterZhao, YingyongLenzi, ErvinZhao, YiwenLéon, Jorge EsquicheZhao, YunLeone, PatriziaZheng, QingbingLeoni, ValerioZheng, WangLeon-Juarez, MoisesZheng, YumeiLeón-Rubio, Jose MaríaZhenwu, LuoLeon-Sicairos, NidiaZhong, ZifuLeow, ChiuanZhou, DaLetoha, TamásZhou, HaoLeung, Ho Man HomanZhou, JiajiaLevy, ItzchakZhou, PanpanLewandowska, AnnaZhou, QianLewis-Ximenez, Lia L.Zhou, WenhaoLeyrat, CedricZhu, BinLi, HeZhu, WenjunLi, JieZicker, FabioLi, JinganZidovec-Lepej, SnjezanaLi, Jin-luanZielinski-Gutierrez, EmilyLi, JunZimmermann, AlbertLi, LiZimmermann, StefanLi, Mei-LingZizza, AntonellaLi, MingZmora, PawelLi, QihanZohlnhöfer, ReimutLi, QiupingZoidis, Ann M.Li, ShaoweiZost, SethLi, XuyongZotti, TizianaLi, YinZou, KeyuanLi, YongkuiZou, PengLian, Ie-BinZuber, PatrickLiang, BinhuaZubiri, LeyreLiang, KaixinZughaier, SusuLiao, Hung-JuZumbrun, ElizabethLiaqat, IramZuñiga, SoniaLiberatore, GiuseppeŻurawski, JakubLichtenwalner, AnneZurriaga, ÓscarLiebman, MichaelZwaveling, JuliëtteLiechti, George W.Zych, DawidLiesveld, JaneŻychowska, MagdalenaLim, Kwang Suk



